# Supplementary material for: Risk Assessment and the Effects of Refuge Availability on the Defensive Behaviors of the Southern Unstriped Scorpion (Vaejovis carolinianus)
Source: Toxins (Basel). 2020 Aug 20;12(9):534. doi: 10.3390/toxins12090534 (PMC7551882; doi:10.3390/toxins12090534)
Supplement: Supplementary file 1 [file toxins-12-00534-s001.pdf]

Supplementary Materials: Risk Assessment and the Effects of Refuge Availability on the Defensive Behaviors of the Southern Unstriped Scorpion (*Vaejovis carolinianus*)

David R. Nelsen, Emily M. David, Chad N. Harty, Joseph B. Hector and Aaron G. Corbit

Table S1. Fixed effects parameter estimates for all Poisson Generalized Linear Mixed Models.

Sting Frequency

					Exp(β) 95% CI			
**Names**	**Effect**	**Estimate**	**SE**	**Exp(β)**	**Lower**	**Upper**	**z**	**p**
(Intercept)	(Intercept)	1.91	0.18	6.76	4.76	9.59	10.71	<0.001
Refuge 1	2 - 0	0.05	0.09	1.05	0.88	1.27	0.57	0.571
Refuge 2	4 - 0	0.15	0.10	1.16	0.95	1.42	1.43	0.152
Prod Location 1	metasoma - chela	0.33	0.08	1.39	1.18	1.64	3.98	<0.001
Prod Location 2	prosoma - chela	0.90	0.08	2.46	2.12	2.85	11.99	<0.001
Sex 1	M - F	0.36	0.67	1.44	0.39	5.38	0.54	0.588
Mean Movement		0.24	0.06	1.27	1.14	1.42	4.20	<0.001
Observation Time		0.01	0.01	1.01	0.99	1.03	0.82	0.414
Scorpion Length		0.13	0.16	1.14	0.83	1.57	0.80	0.426
Refuge 1 × Sex 1	2 - 0 × M - F	−0.44	0.17	0.64	0.46	0.89	−2.64	0.008
Refuge 2 × Sex 1	4 - 0 × M - F	−0.50	0.20	0.61	0.41	0.91	−2.44	0.014

Pinch Frequency

					Exp(β) 95% CI			
**Names**	**Effect**	**Estimate**	**SE**	**Exp(β)**	**Lower**	**Upper**	**z**	**p**
(Intercept)		−2.04	0.39	0.13	0.06	0.28	−5.19	<0.001
Refuge 1	2 - 0	−0.11	0.38	0.90	0.43	1.89	−0.29	0.775
Refuge 2	4 - 0	−1.81	0.76	0.16	0.04	0.73	−2.37	0.018
Prod Location 1	metasoma - chela	−2.23	0.61	0.11	0.03	0.35	−3.68	<0.001
Prod Location 2	prosoma - chela	−0.56	0.31	0.57	0.31	1.06	−1.78	0.076
Sex 1	M - F	−0.12	1.09	0.88	0.10	7.48	−0.11	0.909
Mean Movement		0.19	0.18	1.21	0.85	1.72	1.04	0.297
Observation Time		0.10	0.06	1.10	0.98	1.24	1.58	0.114
Scorpion Length		0.07	0.23	1.08	0.69	1.68	0.32	0.749
Refuge 1 × Sex 1	2 - 0 × M - F	−1.20	0.88	0.30	0.05	1.68	−1.37	0.171
Refuge 2 × Sex 1	4 - 0 × M - F	−0.43	1.53	0.65	0.03	13.01	−0.28	0.778

Venom Use

					Exp(β) 95% CI			
**Names**	**Effect**	**Estimate**	**SE**	**Exp(β)**	**Lower**	**Upper**	**z**	**p**
(Intercept)		−2.08	0.33	0.13	0.07	0.24	−6.25	<0.001
Refuge 1	2 - 0	0.58	0.48	1.78	0.69	4.59	1.19	0.232
Refuge 2	4 - 0	0.15	0.69	1.16	0.30	4.52	0.21	0.833
Prod Location 1	metasoma - chela	−0.15	0.56	0.86	0.29	2.55	−0.28	0.782
Prod Location 2	prosoma - chela	1.01	0.44	2.73	1.15	6.50	2.27	0.023
Sex 1	M - F	−1.53	0.90	0.22	0.04	1.27	−1.70	0.090

Mean Movement		0.57	0.22	1.77	1.15	2.72	2.57	0.010
Observation Time		0.03	0.04	1.03	0.95	1.11	0.64	0.521
Scorpion Length		0.02	0.19	1.02	0.70	1.49	0.09	0.927
Refuge 1 × Sex 1	2 - 0 × M - F	1.62	0.97	5.06	0.75	33.95	1.67	0.095
Refuge 2 × Sex 1	4 - 0 × M - F	−1.14	1.36	0.32	0.02	4.61	−0.84	0.401

Cycle

					Exp(β) 95% CI			
**Names**	**Effect**	**Estimate**	**SE**	**exp(β)**	**Lower**	**Upper**	**z**	**p**
(Intercept)		2.35	0.18	10.43	7.31	14.88	12.94	<0.001
Cycle 1	2 - 1	0.24	0.06	1.27	1.14	1.41	4.30	<0.001
Cycle 2	3 - 1	−0.06	0.06	0.94	0.84	1.06	−0.96	0.339

Table S2. Fixed effects parameter estimates for all Linear Mixed Models.

Flee Duration								
				95% CI				
**Names**	**Effect**	**Estimate**	**SE**	**Lower**	**Upper**	**df**	**t**	**p**
(Intercept)		4.51	0.49	3.55	5.47	15.54	9.24	<0.001
Refuge 1	2 - 0	0.43	0.77	−1.09	1.95	111.9	0.56	0.579
Refuge 2	4 - 0	0.8	0.78	−0.73	2.32	113.32	1.02	0.309
Prod Location 1	metasoma - chela	4.45	0.74	3.01	5.9	104.15	6.03	<0.001
Prod Location 2	prosoma - chela	4.67	0.74	3.21	6.13	104.24	6.28	<0.001
Sex 1	M - F	−1.21	2.24	−5.61	3.19	17.54	−0.54	0.596
Mean Movement		−0.08	0.32	−0.71	0.55	65.75	−0.26	0.797
Observation Time		0.06	0.08	−0.1	0.22	109.16	0.77	0.444
Scorpion Length		−0.17	0.54	−1.23	0.9	16.82	−0.31	0.764
Refuge 1 × Sex 1	2 - 0 × M - F	−2.61	1.54	−5.63	0.4	114.89	−1.7	0.092
Refuge 2 × Sex 1	4 - 0 × M - F	−0.36	1.61	−3.51	2.79	112.35	−0.22	0.824
**Latency to Sting**								
				95% CI				
**Names**	**Effect**	**Estimate**	**SE**	**Lower**	**Upper**	**df**	**t**	**p**
(Intercept)		13.17	2.21	8.84	17.51	2.37	5.96	0.018
Refuge 1	2 - 0	−3.39	2.58	−8.44	1.66	26.4	−1.32	0.199
Refuge 2	4 - 0	−2.58	2.38	−7.23	2.08	25.95	−1.08	0.288
Sex 1	M - F	2.93	5.82	−8.48	14.34	15.59	0.5	0.622
Mean Movement		−2.1	0.9	−3.87	−0.33	37.8	−2.33	0.026
Scorpion Length		1.41	1.4	−1.35	4.16	14.28	1	0.333
Refuge 1 × Sex 1	2 - 0 × M - F	−4.18	4.9	−13.78	5.43	26.47	−0.85	0.402
Refuge 2 × Sex 1	4 - 0 × M - F	0.82	4.7	−8.4	10.04	24.34	0.17	0.863
**Latency to Pinch**								
				95% CI				
**Names**	**Effect**	**Estimate**	**SE**	**Lower**	**Upper**	**df**	**t**	**p**
(Intercept)		69.73	9.06	51.97	87.48	7.67	7.70	< 0.001
Refuge 1	2 - 0	−30.10	11.32	−52.28	−7.92	15.61	−2.66	0.017
Refuge 2	4 - 0	17.13	11.33	−5.08	39.34	14.89	1.51	0.152
Sex 1	M - F	27.07	40.78	−52.85	106.99	15.76	0.66	0.516
Mean Movement		−3.60	5.26	−13.91	6.71	32.68	−0.68	0.499
Scorpion Length		1.51	9.93	−17.96	20.98	15.13	0.15	0.881
Refuge 1 × Sex 1	2 - 0 × M - F	5.14	22.13	−38.24	48.52	18.06	0.23	0.819
Refuge 2 × Sex 1	4 - 0 × M - F	54.82	22.96	9.82	99.81	19.93	2.39	0.027
**Latency to Flee**								
				95% CI				
**Names**	**Effect**	**Estimate**	**SE**	**Lower**	**Upper**	**df**	**t**	**p**
(Intercept)		5.94	0.98	4.01	7.87	1.74	6.04	0.036
Refuge 1	2 - 0	1.61	2.22	−2.74	5.96	32.69	0.72	0.474
Refuge 2	4 - 0	−0.22	2.10	−4.34	3.90	39.36	−0.10	0.918
Sex 1	M - F	−6.07	4.18	−14.26	2.13	38.26	−1.45	0.155
Mean Movement		0.52	0.73	−0.92	1.95	38.41	0.70	0.486
Scorpion Length		−1.42	1.00	−3.38	0.53	38.31	−1.43	0.161
Refuge 1 × Sex 1	2 - 0 × M - F	−2.33	4.36	−10.87	6.21	39.98	−0.53	0.596

Refuge 2 × Sex 1	4 - 0 × M - F	−4.18	4.27	−12.54	4.18	39.11	−0.98	0.333

Mean Movement

				95% CI				
**Names**	**Effect**	**Estimate**	**SE**	**Lower**	**Upper**	**df**	**t**	**p**
(Intercept)		3.31	0.28	2.76	3.86	12.50	11.87	< 0.001
Refuge 1	2 - 0	0.50	0.37	−0.23	1.22	17.58	1.35	0.195
Refuge 2	4 - 0	−0.10	0.38	−0.85	0.64	17.76	−0.27	0.787
Sex 1	M - F	0.36	0.56	−0.73	1.46	12.50	0.65	0.528
Refuge 1 × Sex 1	2 - 0 × M - F	−0.44	0.74	−1.89	1.01	17.58	−0.60	0.559
Refuge 2 × Sex 1	4 - 0 × M - F	0.89	0.76	−0.59	2.38	17.76	1.18	0.255

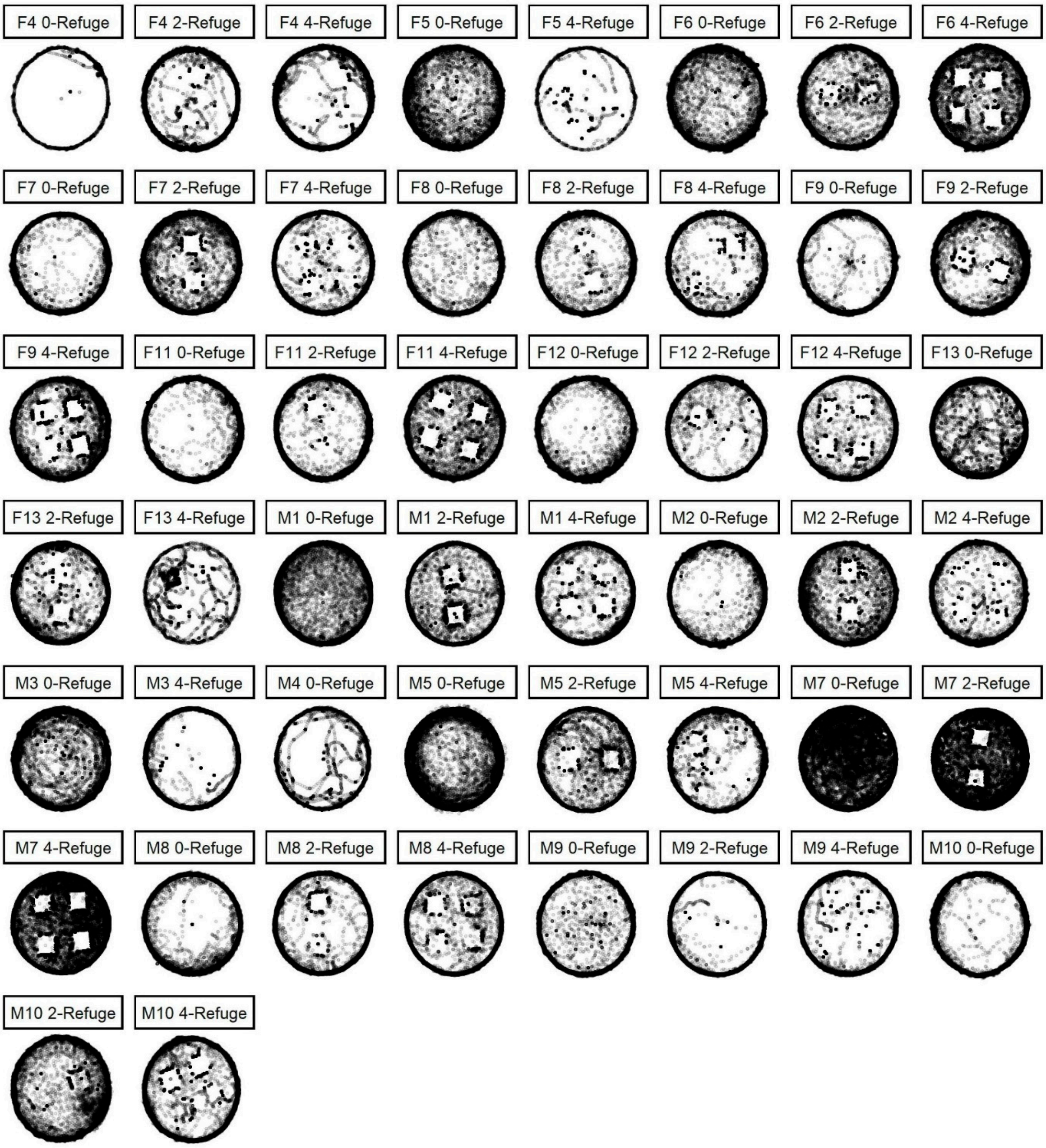

Figure S1. Movements of the scorpion, *Vaejovis carolineanus,* during the overnight acclimation period of 50 experimental trials. Plots are based on X/Y coordinates obtained from video footage using a frame subtraction algorithm in MATLAB. Darker areas represent locations where the scorpion visited more frequently, and white areas represent areas the scorpion was not observed. Labels show scorpion sex (M or F), numeric identifier, and the number of refuges present in the arena.
